# Lipid metabolic disorders and their impact on cartilage endplate and nucleus pulposus function in intervertebral disk degeneration

**DOI:** 10.3389/fnut.2025.1533264

**Published:** 2025-03-10

**Authors:** Ruixia Wu, Xiao Juan Zhao, Yaxin Du, Yizhi Dong, Xinyue Song, Yong Zhu

**Affiliations:** ^1^Inner Mongolia Medical University, Hohhot, China; ^2^The Second Affiliated Hospital of Inner Mongolia Medical University, Hohhot, China; ^3^Peking University Cancer Hospital Inner Mongolia Hospital, Affiliated Cancer Hospital of Inner Mongolia Medical University, Hohhot, China

**Keywords:** lipid metabolism, lipid metabolic disorders, intervertebral disk degeneration, cartilage endplate, nucleus pulposus, oxidative stress

## Abstract

Lipid metabolism encompasses the processes of digestion, absorption, synthesis, and degradation of fats within biological systems, playing a crucial role in sustaining normal physiological functions. Disorders of lipid metabolism, characterized by abnormal blood lipid levels and dysregulated fatty acid metabolism, have emerged as significant contributors to intervertebral disk degeneration (IDD). The pathogenesis of IDD is multifaceted, encompassing genetic predispositions, nutritional and metabolic factors, mechanical stressors, trauma, and inflammatory responses, which collectively facilitate the progression of IDD. Although the precise mechanisms underlying IDD remain incompletely elucidated, there is substantial consensus regarding the close association between lipid metabolism disorders and its development. Intervertebral disks are essential for maintaining spinal alignment. Their primary functions encompass shock absorption, preservation of physiological curvature, facilitation of movement, and provision of stability. The elasticity and thickness of these disks effectively absorb daily impacts, safeguard the spine, uphold its natural curvature and flexibility, while also creating space for nerve roots to prevent compression and ensure normal transmission of nerve signals. Research indicates that such metabolic disturbances may compromise the functionality of cartilaginous endplates (CEP) and nucleus pulposus (NP), thereby facilitating IDD’s onset and progression. The CEP is integral to internal material exchange and shock absorption while mitigating NP herniation under mechanical load conditions. As the central component of intervertebral disks, NP is essential for maintaining disk height and providing shock-absorbing capabilities; thus, damage to these critical structures accelerates IDD progression. Furthermore, lipid metabolism disorders contribute to IDD through mechanisms including activation of endoplasmic reticulum stress pathways, enhancement of oxidative stress levels, induction of cellular pyroptosis alongside inhibition of autophagy processes—coupled with the promotion of inflammation-induced fibrosis and fibroblast proliferation leading to calcification within intervertebral disks. This review delineates the intricate interplay between lipid metabolism disorders and IDD; it is anticipated that advancing our understanding of this pathogenesis will pave the way for more effective preventive measures and therapeutic strategies against IDD in future research.

## 1 Introduction

**Figure 1:**
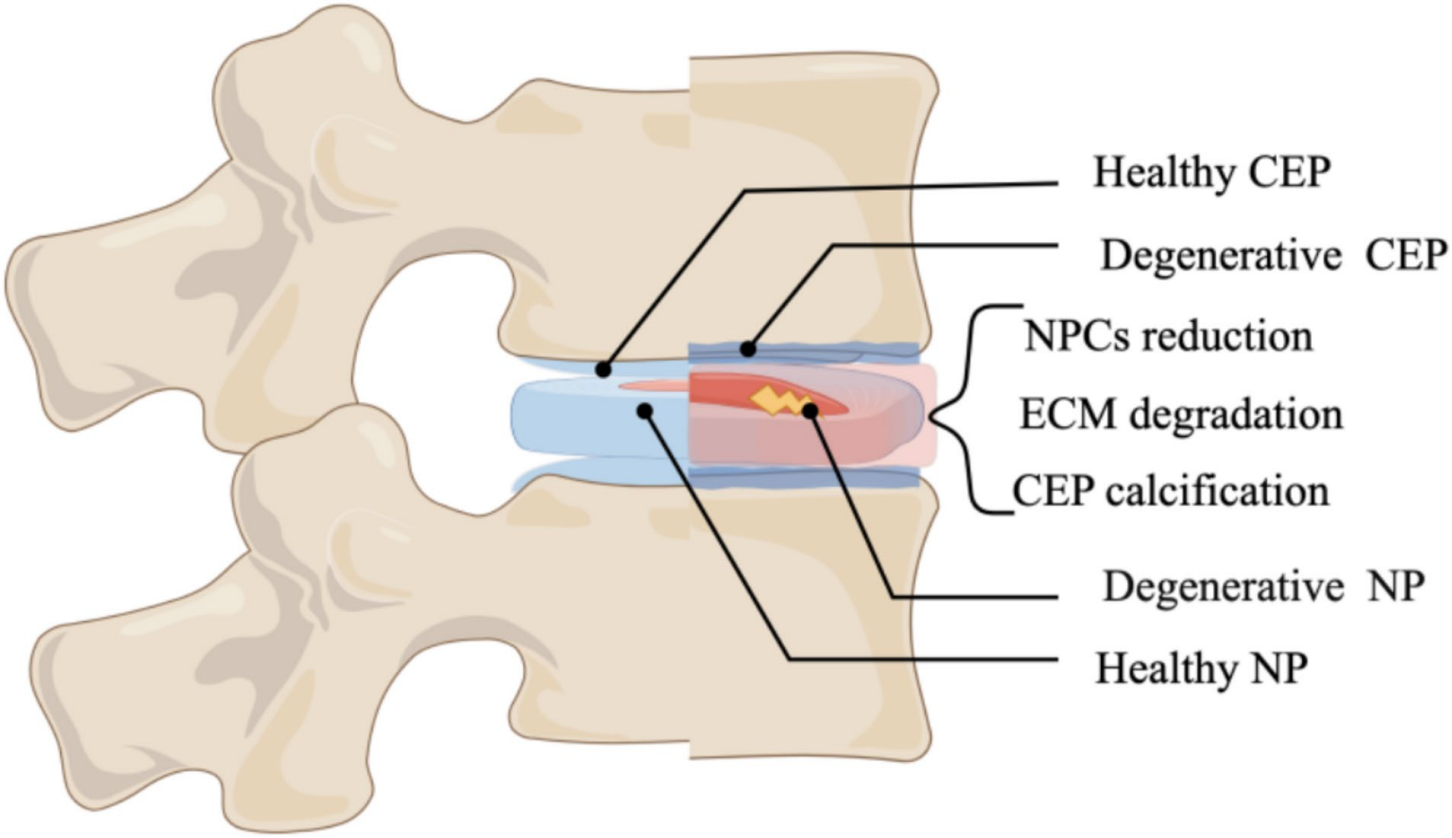
Illustrates the primary manifestations of intervertebral disk degeneration (By Figdraw).

IDD is characterized by a multifaceted process of aging and damage to the intervertebral disk (IVD), driven by intricate molecular mechanisms, which ultimately culminate in significant clinical manifestations (1). As illustrated in Figure 1, the primary manifestations encompass a reduction in the number of nucleus pulposus cells (NPCS), dysregulation of extracellular matrix metabolism, diminished levels of type II collagen, calcification, and apoptosis of endplate chondrocytes, among others (2) (Figure 1). Low back pain (LBP) is a hallmark clinical manifestation of IDD and represents one of the primary contributors to global productivity loss (3). It has been reported that 26 to 42% of IDD patients experience LBP (4), LBP continued to be the predominant contributor to disability-adjusted life years (YLDs) on a global scale. Lower back pain and the associated YLDs escalate as individuals age, peaking around age 85. Notably, across all age demographics, the prevalence of lower back pain is significantly higher among women than men (5). Despite the significant prevalence and severity of LBP, there remains a lack of curative or effective disease-modifying therapies, primarily due to an insufficient understanding of its underlying pathogenesis. IDD is thought to arise from a multifactorial interplay of biomechanical stress, metabolic disturbances, and nutritional deficiencies; nevertheless, the precise pathological mechanisms underlying IDD are still inadequately defined (6, 7). Lipid metabolism encompasses the processes of digestion, absorption, synthesis, and degradation of fats, mediated by various enzymes within the body. The metabolic products include lipid mediators, fatty acids, and cholesterol derivatives. Under physiological conditions, lipid mediators play a crucial role in human growth and development, metabolic regulation, and tissue remodeling (8). Dysregulation of lipid metabolism has been implicated in the pathogenesis of obesity, hyperlipidemia, and hypercholesterolemia (9). Research indicates a multifaceted relationship between dysregulated lipid metabolism and IDD (10). Factors influencing lipid metabolism, such as body weight, body mass index (BMI), and blood lipid levels, significantly correlate with IDD progression. Notably, elevated triglyceride (TG) levels have been identified as a critical risk factor for IDD. In individuals with obesity, the prevalence of hypertriglyceridemia is markedly increased, and hyperlipidemia has been linked to cellular metabolic dysfunction and inflammation in disk cells (11).

A recent study investigating the influence of obesity on IDD has identified CXCL16 as a potential diagnostic biomarker for both obesity and IDD, highlighting its role in regulating fatty acid metabolism and facilitating IDD progression (12). In murine models of degenerative intervertebral disk disease, more than 50 lipid metabolites have been identified that exhibit significant expression differences (13). Preliminary investigations have identified CYP27A1, FAR2, and CYP1B1 as promising diagnostic biomarkers, further substantiating their critical involvement in the pathogenesis of IDD associated with lipid metabolism (14). In recent years, researchers have conducted extensive investigations into the regulatory mechanisms underlying lipid metabolism disorder signals and their impact on the aging and calcification of intervertebral disk endplate chondrocytes (EPCs). Their findings indicate that oxidized low-density lipoprotein (ox-LDL) and its receptor LOX-1 activate the ROS/P38-MAPK/NF-κB signaling pathway, promoting EPC senescence and calcification. This work offers novel insights into the intricate relationship between lipid metabolism disorders and IDD (10). Although lipid metabolism disorders are recognized as significant contributors to the pathogenesis of IDD, the precise mechanisms underlying their effects remain inadequately understood. This review aims to provide a comprehensive discussion on the role of lipid metabolism disorders in IDD development and to investigate their potential mechanistic pathways further.

## 2 An overview of the structure and function of the intervertebral disk

The IVD is a complex structure situated between adjacent vertebrae, comprising three interrelated tissues: NP, AF, and CEP. The NP functions as the central component of the IVD and is characterized by its highly hydrated, gel-like consistency, which contains predominantly type II collagen fibers (15). NP cells modulate the metabolic processes of the extracellular matrix (ECM) by synthesizing proteoglycans and collagen. The ECM predominantly comprises type II collagen fibers and proteoglycans, including aggrecan and versican. Type II collagen fibers confer essential mechanical strength to NP, while glycoproteins with negatively charged side chains preserve tissue volume and shape through their remarkable water-binding capacity, thereby endowing NP with exceptional elasticity and swelling properties due to water absorption. This structural integrity is crucial for maintaining intervertebral disk height and alleviating pressure, thus safeguarding the vertebrae and spinal cord from potential injury (16, 17). As individuals age or experience degenerative changes, the ECM of the IVD undergoes a loss of components and structural degradation, resulting in diminished disk height and functional impairment. Consequently, maintaining an optimal balance between collagen fibers and proteoglycans within NP is essential for preserving IVD health and functionality. The annulus fibrosus (AF), which encases NP, constitutes an organized structure comprising several adjacent concentric layers of collagen (18). This structure endows the AF with favorable mechanical properties when subjected to compressive loading (18). In particular, the outer layer exhibits a type I collagen content as high as 95%, which imparts essential tensile strength to IVD. However, transitioning toward the inner layer and approaching the nucleus, there is a marked reduction in type I collagen content, dropping to less than 5%. Conversely, type II collagen content gradually diminishes as it nears the outer layer of the annulus fibrosus. This specific structural arrangement facilitates tensile resistance that limits the radial expansion of the nucleus under compressive loading conditions, thereby aiding in maintaining normal physiological curvature of the spine (15, 19). Furthermore, the IVD represents the largest avascular tissue structure in the human body, with cellular material exchange predominantly occurring through diffusion via microscopic pores on its end plates. This distinctive physiological characteristic renders the IVD particularly vulnerable to degenerative changes (1). The endplate is a bony-cartilaginous structure composed of two distinct components: the CEP and the bony endplate (BEP). These components physically confine NP and annulus fibrosus (AF) within their anatomical confines. Beyond its mechanical support function, the CEP, characterized by a dense layer of type II collagen-rich hyaline cartilage, anchors IVD to adjacent vertebral bodies. Moreover, the CEP plays a crucial role in regulating fluid exchange and nutrient and metabolic waste transport. Acting as a semipermeable barrier between the IVD and vertebral bodies, it facilitates nutrient transfer from nearby blood vessels to the IVD, providing essential nutritional support for its maintenance (17). In conclusion, the intervertebral disk is vital to preserving the stability, flexibility, and protection of the spinal cord and nerve roots. Any injury or degenerative alterations to the intervertebral disk can result in functional impairments of the spine, potentially leading to a spectrum of spine-related pathologies. Consequently, ensuring the health of the intervertebral disk is vital for preventing spinal disorders and promoting overall well-being.

## 3 The etiology and pathological progression of IDD

**Figure 2:**
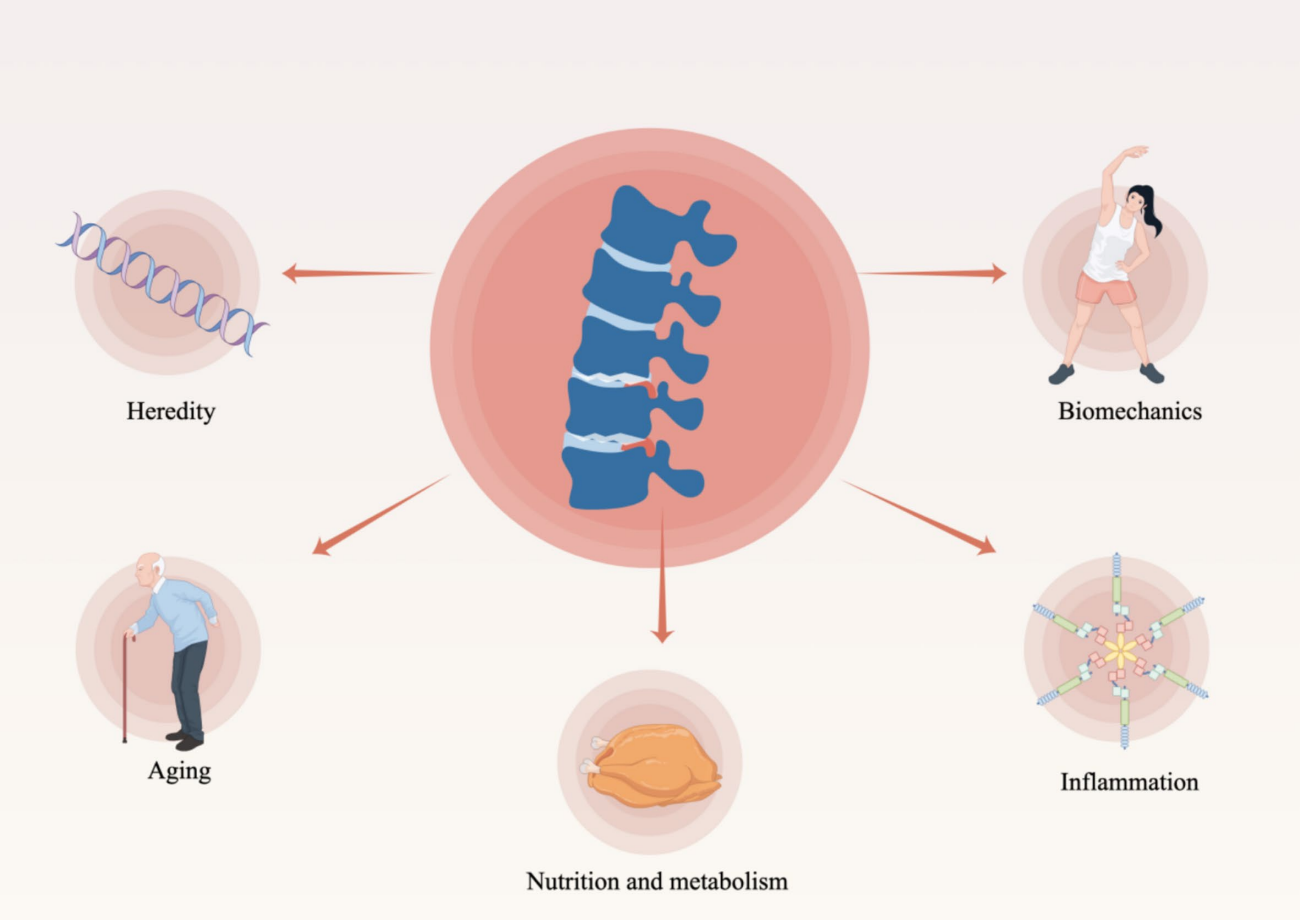
Factors contributing to degenerative alterations in intervertebral disks (By Figdraw).

The IDD represents a multifaceted pathological process characterized by the interplay of various factors and stages. Although the precise mechanisms underlying IDD remain incompletely understood, The pathogenesis of the condition is influenced by a multitude of factors, as illustrated in Figure 2. These factors encompass genetic predisposition, nutritional influences, mechanical stressors, traumatic events, inflammatory responses, cellular senescence, and degenerative processes, all of which significantly contribute to the development and progression of the disease (6, 20) (Figure 2). These factors contribute to an imbalance between the degradation and synthesis of the intervertebral disk, resulting in the deterioration of the extracellular matrix and a subsequent loss of function (6). The following is a brief overview of the main causes of IDD and its pathological changes.

### 3.1 Genetic factors

The primary biological function of IVD is to connect adjacent vertebrae and transmit loads throughout the spine while serving as a critical shock absorber between vertebrae. In the normal aging process, the shape and structure of the intervertebral disk gradually deteriorate, which is traditionally believed to be caused by the cumulative effects of epigenetic, metabolic, and environmental factors (21). Recent genetic studies have elucidated that the pathogenesis of IDD may be influenced by many genetic markers and susceptibility genes, which potentially interact with environmental and lifestyle factors. For instance, polymorphisms in genes encoding proteoglycans, such as ACAN, have been associated with IDD. Regarding disk herniation and IDD, research has indicated that genetic polymorphisms impacting the extracellular matrix components of fibrocartilaginous cell, such as COL11A1, may play a significant role in IDD (22–25). Mice harboring a mutation in the Col9a1 gene display progressive IDD characteristics, potentially attributable to compromised synthesis or assembly of the non-fibrillar Col9a1 chain (26); polymorphisms in the COL9A2 and COL9A3 genes have been associated with sciatica and IDD within the Finnish population (27, 28). Recently, Li et al. (14) conducted a comprehensive investigation into the role of lipid metabolism-related genes in immune infiltration processes, revealing that CYP27A1, FAR2, and CYP1B1 are pivotal lipid metabolism-associated genes significantly contributing to the pathogenesis of IDD. Furthermore, several studies have investigated the genetic correlation between lower back pain and IDD. These investigations have revealed that the genetic contributions to lower back pain and IDD overlap by a proportion ranging from 7 to 23% (29, 30). The estimated heritability of progressive changes in IDD over 5 years varies from 47 to 66% (31). In evaluating the genetic underpinnings of severe IVD, it has been determined that heritability in the lumbar region is approximately 64%, while in the cervical region, it reaches as high as 79% (32). These findings suggest that genetic factors significantly contribute to the pathogenesis and manifestation of disk disease.

### 3.2 Nutritional and metabolic factors

The intervertebral disk is positioned within a closed environment that lacks direct blood supply, and its material exchange with the external environment is exclusively facilitated through the cartilage endplates of adjacent vertebrae (33). IDD is intricately linked to the calcification and hypertrophy of the cartilage and bone endplate (34); this reduction in efficiency diminishes the transport of nutrients and other bioactive factors to AF and NP (34, 35). In normal cartilage endplates, the three-dimensional network of type II collagen is essential for restraining the excessive expansion of NP, thereby preserving the relative stability of the tissue architecture. This structural characteristic mitigates the outward diffusion of water within NP and facilitates nutrient transfer from the vertebral body to the IVD (36). Nonetheless, daily spinal movements can induce microscopic damage to the cartilage endplate and the fibrocartilage ring, leading to degenerative alterations and calcification of the cartilage endplate. This process may obstruct its porous structure, thereby hindering the transport of nutrients from external sources to NP (18). Therefore, the imbalance in material exchange resulting from the degeneration of the cartilage endplate can lead to metabolic disorders in the cells within NP, which are responsible for maintaining the homeostasis of the collagen matrix. This shift toward a fibrotic phenotype results in the loss of water within NP, impairs the load-bearing capacity of the nucleus, reduces disk height, and ultimately leads to IDD (18). Wong et al. (37) demonstrated that elevated levels of collagen, sulfated glycosaminoglycans (sGAGs), and minerals within the extracellular matrix can hinder nutrient diffusion, consequently impacting the viability and functionality of NP cells. Recent reports indicate that identical defects in CEP components are significantly associated with more severe disk degeneration in patients with lower back pain (38). Additionally, Yin et al. (39) obstructed the nutrient supply pathway to the endplate in ovine vertebral models through cement injection. Imaging results obtained at 48 weeks indicated that disk height was significantly reduced, and degeneration was markedly more pronounced in the group with the obstructed nutrient supply pathway compared to the normal control group. Building upon these studies, Habib et al. (33) introduced the novel concept of modifying the extracellular matrix of the entire intervertebral disk under conditions of compressive loading for the first time. Their research revealed a reduction in sGAG content by 33.5% in human samples and 40% in bovine samples within enzyme-treated extracellular matrix intervertebral disks. This finding underscores the potential to enhance nutrient transport—such as cholesterol—within the intervertebral disk through extracellular matrix modification, offering a promising new strategy for addressing IDD.

The studies mentioned above collectively indicate that nutritional and metabolic factors are pivotal in the progression of IDD. Consequently, ensuring an adequate supply of nutrients to the intervertebral disk is essential for delaying the onset of IDD.

### 3.3 Biomechanical factors

The intervertebral disk is crucial in preserving spinal flexibility while facilitating the transfer of compressive forces between adjacent vertebrae. Typically, the intervertebral disk endures loads resulting from flexion and extension, rotation, and various other movements (40). Nevertheless, persistent degenerative changes at the organizational, cellular, and molecular levels can substantially modify the disk’s morphology and physiological properties, diminishing its capacity to endure compressive forces (41). In recent years, there has been a growing recognition of the significance of biomechanical factors in IDD. The IVD, as a distinct closed biomechanical entity, comprises substructures with varying mechanical properties; any impairment to the mechanical integrity of these substructures can adversely affect the overall mechanical functionality of the IVD (42). Prior research demonstrated that abnormal mechanical loading on the spine accelerated the degradation of the disk matrix, promoted neural invasion, induced pyroptosis, and ultimately contributed to IDD (43).

At the movement level, flexion, extension, and rotation of the spine may alter the microstructural components of intervertebral disks—such as collagen, elastin, and proteoglycans—thereby ultimately affecting stress distribution within degenerative intervertebral disks (44). Two critical factors contributing to IDD are the length and magnitude of axial compressive forces. An increase in the magnitude of axial compressive loads results in heightened strain curvature within the posterior lateral region of the IVD; however, this also leads to reduced compliance, hindering the reorientation of its fibrous structure and causing strain amplitude to remain relatively constant. As loading intensifies, the posterior lateral region becomes increasingly vulnerable to stress recruitment, thereby elevating injury susceptibility and potentially facilitating the progression of IDD (45). During flexion, the strain magnitude in the posterior lateral region of the AF significantly increases, with tensile forces in the AF fibers becoming concentrated at the junction between the posterior lateral fiber structure and the endplate. When fully flexed forward, fibers in this region undergo stretching, curling, and straightening, resulting in a flattened configuration that extends to approximately 50–90% of their original length—nearly achieving a vertical orientation—which renders them highly susceptible to substantial pressure (46). This suggests that the development of bony spurs at the periphery of vertebral bodies and lesions at the interface between endplates and annulus fibrosus in IDD is more pronounced in regions experiencing stress concentration. Furthermore, stress distribution appears to be increasingly localized toward the margins of the intervertebral disk, particularly when areas of diminished intervertebral disk height coincide with zones of axial stress concentration (47). Furthermore, Xu et al. (48) employed a three-dimensional finite element model to investigate the alterations in stress within the intervertebral disk during spinal rotation, revealing that the deformation strain of the intervertebral disk exhibits directional characteristics during axial rotation. Under physiological loading conditions, the deformation pattern of the intervertebral disk during lateral rotation is nearly symmetrical. The maximum compressive strain is observed in the intervertebral disk’s posterior region opposite the rotation direction. In contrast, maximal tensile strain occurs in its anterior region aligned with the direction of rotation, specifically at the junction between ligamentum flavum and cartilage endplate. Consequently, during axial rotation of the spine, the posterior lateral region of the intervertebral disk experiences elevated compressive stress under axial loading conditions, particularly in scenarios involving greater compressive forces, which may represent a significant risk factor for IDD.

At the cellular level, loading conditions are pivotal in the pathogenesis of IDD. Mechanical stimuli profoundly influence cells’ physiology, indicating a reciprocal relationship between mechanical and biochemical factors that enhances their synergistic effects (8). Moreover, these factors can modify the cellular microenvironment by altering pH levels, hydration status, and permeability, subsequently impacting cell viability. Additionally, the phenotype and behavior of IVD cells are also modulated by mechanical stimuli (49). The investigation revealed that intermittent cyclic mechanical tension activates the canonical Wnt signaling pathway and the E-cadherin/β-catenin complex, thereby facilitating the degeneration of intervertebral disk endplate cartilage (50). Intermittent cyclic loading similarly induces morphological alterations in disk endplate cells, transitioning from polygonal to elongated shapes. Over time, the expression levels of type II collagen, aggrecan, and SOX-9 in these cells exhibit a time-dependent decline, whereas those of type I collagen, X-type collagen, and osteocalcin show an increase. These findings indicate that intermittent cyclic loading can directly promote the degeneration of disk endplate chondrocytes, resulting in the downregulation of chondrogenic gene expression alongside the upregulation of osteogenic gene expression (35). In summary, comprehending and modulating the biomechanical environment is crucial for preventing and managing IDD.

### 3.4 Other factors

In addition to the factors mentioned above, the progression of IDD is influenced by a variety of other elements, including inflammation, smoking, aging, and trauma, each contributing to the pathological processes associated with IDD through distinct mechanisms. In the pathological process of IDD, inflammatory factors, including tumor necrosis TNF-α, IFN-γ, and various interleukins, play a key role, particularly in promoting inflammatory responses (51). Chen et al. (52) revealed a positive feedback loop involving the activation of the IL-3β/NF-κB-NLRP1 inflammasome, where IL-1β stimulates myeloid cells. This is the first time it has been recorded that IL-1β increases NLRP3 inflammasome activity to aggravate IDD and promotes its expression in response to stimulation. Furthermore, a retrospective study showed that TNF-α not only accelerates the degeneration of the intervertebral disk but also exacerbates pain, with its concentration being positively correlated with the degree of degeneration (53). Recent research has elucidated the detrimental effects of unhealthy lifestyle choices, including obesity, alcohol misuse, and smoking, on human health. Notably, these adverse behaviors are associated with an elevated risk of inflammatory degenerative diseases, including IDD. Staszkiewicz et al. (54) highlighted that the degenerative process of IDD is influenced by lifestyle variations, which in turn affect the concentrations of neurotrophic factors NT-3 and NT-4, thereby facilitating the progression of IDD. Finally, the investigation conducted by Hutchinson et al. (55) examined the relationship between age and IDD. The results indicated that a comparative analysis of the lumbar spines of 6-month-old and 12-month-old mice revealed more pronounced degenerative alterations in the latter group. This study underscores that age is a critical determinant influencing both the progression and severity of IDD.

## 4 The association between lipid metabolic disorders and IDD

### 4.1 The implications of lipid metabolic disorders in IDD

Lipid metabolism encompasses digestion, absorption, synthesis, and degradation of fats in living organisms facilitated by various enzymes. The metabolic products generated through this pathway include lipid mediators such as leptin, adiponectin, and progranulin (56), along with fatty acids and cholesterol derivatives (8). Leptin, a hormone secreted by adipose tissue, exhibits a positive correlation with body weight and plays a crucial role in the pathogenesis of IDD (57). Previous studies have investigated the relationship between leptin and IDD using bovine intervertebral disk model systems. The findings indicate that leptin alone has a relatively minor effect on cellular energy metabolism; however, within a pro-inflammatory environment, leptin demonstrates significant synergistic effects with cytokines, particularly IL-6. This interaction markedly enhances the production of NO, MMPs, and the expression of pro-inflammatory cytokines, thereby facilitating the progression of IDD (58). Similarly, Chen et al. examined the relationship between single nucleotide polymorphisms (SNPs) of the leptin gene (LEP) and plasma leptin levels in patients suffering from IDD. They also analyzed how gender and obesity status influenced this association. The study included a total of 303 Taiwanese IDD patients and was the first to demonstrate a significant correlation between LEP gene polymorphisms and leptin levels specifically in obese women with IDD. Leptin is known to play a crucial role in the degenerative processes affecting intervertebral disks by promoting inflammatory responses and facilitating extracellular matrix degradation. However, further research is necessary to validate these findings across larger sample sizes and diverse ethnic populations (59). Adiponectin is an adipokine that exhibits dysregulation in obesity and plays a significant role in various pathological processes, including degenerative disk disease (60). Bin et al. (61) reported a decrease in the expression levels of adiponectin in IDD and NPCs. Furthermore, adiponectin has been shown to inhibit the expression of TNF-α in degenerated NPCs, thereby potentially delaying IDD. Recently, Hiroki et al. (62) discovered that the adiponectin receptor agonist AdipoRon can protect IVD from degeneration both *in vivo* and *in vitro*. Numerous studies have demonstrated that Progranulin (PGRN) exerts anti-inflammatory effects in both Rheumatoid Arthritis (RA) and Osteoarthritis (OA) (63). Elevated levels of PGRN are correlated with the amelioration of clinical symptoms. In a murine model of IDD, the absence of PGRN accelerates the degenerative process, as evidenced by an increase in osteoblast markers, depletion of proteoglycans, and upregulation of inflammatory mediators (64). The involvement of PGRN in IDD may be linked to its regulation of cytokines such as IL-10 and IL-17, potentially mediated through TNFR1 and TNFR2 (65). Notably, engineered variants of PGRN are anticipated to inhibit the degeneration process induced by TNF-α, indicating that PGRN represents a promising therapeutic target for IDD, which necessitates further investigation in human studies (66). Jianye et al. (67) demonstrated that the activity of the arachidonic acid metabolic pathway is positively correlated with the severity of IDD, and this metabolic activity is significantly enhanced in macrophages and neutrophils. Xiang et al. (68) investigated the role of n-3 polyunsaturated fatty acids (PUFAs) in IDD and its underlying mechanisms. By establishing IDD models in both wild-type (WT) and transgenic (TG) mice, the study demonstrated that TG mice exhibited an increased content of n-3 PUFAs and a reduced *n*−6/*n*−3 PUFAs ratio, which significantly decelerated the progression of IDD as well as the aging process of intervertebral disk cells.

Lipid metabolic disorders are widely recognized as a significant contributor to the onset and progression of various diseases, including cancer, type 2 diabetes, and osteoarthritis (69). Additionally, lipid metabolic disorders may contribute to the progression of atherosclerosis, resulting in diminished blood flow to the lumbar region and consequently heightening the risk of IDD, sciatica, and lower back pain (70, 71). Research indicates that elevated cholesterol levels may increase the risk of spinal degenerative disorders, including lumbar degeneration (72, 73). This may be attributed to obesity or being overweight (74). A case–control study conducted in China revealed that the ratios of total cholesterol (TC) to high-density lipoprotein cholesterol (HDL-C) and low-density lipoprotein cholesterol (LDL-C) to HDL-C were significantly associated with disk herniation. Furthermore, patients exhibiting elevated serum LDL-C levels demonstrated an increased risk of developing disk herniation, indicating that serum lipid profiles may serve as valuable predictors of disk degeneration within the Chinese population (75). Shortly thereafter, a comprehensive cross-sectional survey conducted among Japanese adults aged 40–64 residing in a foreign country revealed an association between HDL-C levels and the ratio of LDL-C to HDL-C with lower back pain (76). Similarly, Liang et al. conducted a comprehensive analysis of the influence of lipid metabolic disorders on IDD and arrived at significant conclusions. Their findings indicate that elevated cholesterol levels in the bloodstream play a pivotal role in the pathogenesis of IDD, thereby underscoring the potential association between lipid metabolic disorders and IDD (77). A study investigating the severity of disk degeneration revealed that elevated triglyceride (TG) levels and abdominal obesity exert a more pronounced influence on the extent of disk degeneration, suggesting their pivotal role in the pathogenesis of IDD. Nonetheless, this investigation was constrained by a limited patient cohort and focused exclusively on phenotypes characterized as “healthy lipid but obese” and “dyslipidemic but not obese” (78). Recently, Huang et al. (72) conducted a comprehensive evaluation of the association between serum lipid levels and the extent of IDD, revealing that age, high-density lipoprotein, and triglycerides significantly influence the degree of degeneration in patients with symptomatic lumbar degenerative disk disease who do not have underlying health conditions.

### 4.2 The impact of lipid metabolic byproducts on the functionality of CEP

The CEP is the primary conduit for nutrient delivery and metabolite exchange within the IVD (79). Previous research has demonstrated that a significant and independent decline in the CEP is associated with IDD, indicating that this decline may be a primary factor in the pathogenesis of IDD (80). Moreover, calcification of the CEP compromises its roles in nutrient transport, pressure regulation, and metabolite exchange within the intervertebral disk, thereby accelerating the onset and progression of IDD. Additionally, CEP near blood vessels may be an early indicator or be affected by lipid metabolism disorders (4).

Cartilage tissue is widely recognized for its high content of saturated fatty acids, which play a critical role in preserving its structural integrity and physiological functionality (81). Disruptions in lipid composition or metabolic processes can compromise the functional capacity of cartilage (81). Pathological calcification of cartilaginous tissue results in irreversible degeneration of the CEP, thereby obstructing the material exchange pathway between the intervertebral disk and the vertebral vein. This obstruction leads to a continuous deterioration of the internal metabolic environment within the intervertebral disk, consequently accelerating its degeneration. Li et al. (82) identified a ferritin-like iron-binding protein known as LTF, demonstrating a marked reduction in expression within degenerated human and rat CEP tissues. The downregulation of LTF is associated with enhanced calcification, accelerated aging, and degradation of the extracellular matrix in human chondrocytes.

Furthermore, an animal study has demonstrated that cholesterol is crucial in facilitating the differentiation and maturation of chondrocytes (83). As a critical component of cell membranes, cholesterol plays a pivotal role in maintaining membrane function and fluidity; thus, any alteration in its levels may disrupt these properties, potentially resulting in aberrant cellular behavior and excessive cholesterol accumulation (84). Consequently, hypercholesterolemia could facilitate lipid oxidation and deposition within tissues, ultimately contributing to cartilage degeneration (85). A comprehensive examination of risk and lifestyle factors in patients with degenerative spinal diseases indicates that elevated levels of low-density lipoprotein cholesterol and triglycerides are correlated with a heightened risk of disk herniation (86). Oxidized low-density lipoprotein (ox-LDL) is a product of lipid peroxidation derived from the oxidative modification of low-density lipoprotein (LDL), which serves as a significant pathogenic factor in lipid metabolism disorders (87, 88). Tang et al. thoroughly investigated the effects of lipid metabolism disorders, particularly hyperlipidemia (HLP), on IDD. Their systematic analysis confirmed that HLP contributes to the degeneration of cartilage endplates and enhances the expression of oxidized low-density lipoprotein (ox-LDL) and its receptor LOX-1, thereby promoting both aging and calcification of the CEP, which ultimately facilitates the progression of IDD in the context of persistent lipid metabolism disturbances (10).

In addition to the studies above, certain metabolic byproducts of lipids have been shown to exert specific effects on the cartilage end plate, as reported by Chen et al. (85). Their research indicates that under conditions of estrogen deficiency, these byproducts can promote osteoclastogenesis, resulting in pathological alterations in the cells of the cartilage end plate. In the investigation conducted by Zhang et al. (89), the effects of melatonin on apoptosis and calcification in terminal cells were thoroughly examined. The findings indicated that melatonin intervention significantly reduced the apoptosis rate in terminal cells and inhibited cell calcification, thereby decelerating IDD progression. The research team led by Han et al. (90) investigated the influence of leptin on calcification of the CEP. By developing a rat model of IDD, they found that during CEP cell calcification, there was a significant increase in leptin expression levels, which closely correlated with the progression of IDD. This study indicates that leptin may facilitate CEP cell calcification. In a survey conducted by Hua et al. (91), a positive correlation was observed between IDD and the levels of matrix metalloproteinase-1 (MMP-1) and leptin. Subsequent investigations further elucidated the effects of leptin on MMP-1 expression in human intervertebral disk chondrocyte-derived stem cells, particularly SV40 cells, along with its underlying mechanisms. The findings revealed that leptin activated the RhoA/ERK1/2/NF-kB signaling pathway, thereby enhancing MMP-1 expression in SV40 cells and facilitating the progression of IDD.

The collective findings of this research underscore the pivotal role of lipid metabolism and its derivatives in preserving cartilage tissue health, including that of the cartilage endplate. They also elucidate a potential association between dysregulation of lipid metabolism and IDD. This discovery offers significant insights for further investigation into the implications of aberrant lipid metabolism in the pathogenesis of IDD.

### 4.3 The impact of lipid metabolic byproducts on the functionality of NP

The unique structural composition of NP endows it with exceptional elasticity and a remarkable capacity for water absorption and swelling, which are crucial for preserving the height of intervertebral disks and providing cushioning against mechanical stress, thereby safeguarding the vertebrae and spinal cord from potential injury (16, 17). In the pathological progression of IDD, the excessive aging and apoptosis of NPCs are pivotal factors. Cholesterol, a crucial byproduct of lipid metabolism, has been extensively acknowledged for its significant influence on the pathogenesis of degenerative disk diseases. Apolipoprotein E (ApoE), a structural protein associated with high-density lipoprotein, is essential in maintaining cholesterol homeostasis (92). Zhou et al. (93) found that knocking out ApoE promoted apoptosis of NP and AF cells, ECM degradation, and calcification of CEP. Cholesterol levels were elevated in both human and rat degenerative NP tissues. Their *in vitro* studies showed that cholesterol loading upregulated the pro-cholesterol-regulating element-binding protein 1 (SREBP1) expression, leading to endoplasmic reticulum stress. They found that knocking out SREBP1 weakened cholesterol-induced NP cell apoptosis and restored the production of aggrecan and type II collagen, which helped slow down the progression of IDD (94). Recent research has demonstrated that, alongside apoptosis, pyroptosis represents a critical form of cell death implicated in IDD (95). Research indicates that the concentration of apoptotic cell death markers in degenerative intervertebral disk tissue is significantly elevated compared to normal intervertebral disk tissue (96).

Moreover, SD rats subjected to a high-cholesterol diet exhibited more pronounced IDD compared to those on a normal diet. Recent studies have confirmed that the degeneration of intervertebral disks is intricately linked to the onset of pyroptosis in NP cells. This form of pyroptosis is characterized by dysregulated fatty acid metabolism within NP cells under degenerative pathological conditions, while an inflammatory microenvironment and extracellular matrix degradation further exacerbate this process (97). To address this issue, Wang developed a composite of fibrinogen hydrogels (FG@PEV), which effectively modulates the abnormal fatty acid metabolism in NPCs by regulating the synthesis and degradation of the extracellular matrix. This intervention prevents cell pyroptosis and mitigates disk degeneration (97). It has been reported that the expression levels of adiponectin, a fatty factor with anti-inflammatory properties, are significantly downregulated in the degenerated NP of humans. The study conducted by Wu et al. corroborated this finding, demonstrating that adiponectin can effectively inhibit lipopolysaccharide-induced apoptosis in NPCs (98). Chen et al. underscored the pivotal role of palmitic acid (PA) in the pathogenesis and disease-associated phenotypes of IDD. Their findings revealed a significant accumulation of lipid droplets in the nuclei of NPCs in late-stage IDD samples. Subsequent investigations indicated that the abnormal buildup of PA in IDD-affected spinal cord cells resulted in lipid droplet formation and cellular senescence, which is attributable to prolonged PA exposure (99). Under physiological conditions, serum levels of leptin are directly proportional to body fat storage. By binding to its receptors (leptin receptor, LepR) on target cells, leptin activates associated signaling pathways that play a crucial role in processes such as inflammation and cellular metabolism (100). Gao et al. (101) investigated NP cells derived from surgical specimens of 20 patients suffering from degenerative disk disease or scoliosis, with an average age of 36 years. Their findings revealed a significant decline in the proportion of leptin receptor-positive (LepR-positive) cells with increasing degrees of disk degeneration, from 75% at grade II to 32% at grade V, according to the Pfirrmann classification.

Additionally, obesity has been demonstrated to facilitate IDD through non-mechanical pathways, with the underlying mechanism involving alterations in free fatty acid concentrations that may adversely affect cellular metabolism (11). Zhang et al. (11) conducted a retrospective study to review the IDD severity of 128 volunteers. They compared it with obesity-related factors, including body weight, body mass index (BMI), and serum lipid levels. The study found that volunteers in the IDD group had increased age, BMI, and serum triglyceride levels. Further animal experiment results showed that obesity aggravates the development of IDD by activating the MAPK signaling pathway, leading to increased apoptosis of NPCs and imbalanced extracellular matrix metabolism. The global trend of physical inactivity has contributed to a rising incidence of diseases such as obesity and diabetes (102, 103). To address this issue, Zhengqi et al. (104) examined the interaction between exercise and IDD. Their study revealed that Meteorin-like protein (Metrnl) (105), a newly identified muscle-derived factor involved in lipid metabolism regulation, was upregulated in the muscles, serum, and NP tissue of exercised rats. Specifically, Metrnl enhances lipid utilization in NPCs by activating peroxisome proliferator-activated receptor α (PPARα), which subsequently activates carnitine palmitoyltransferase 1A (CPT1A), the rate-limiting enzyme in fatty acid β-oxidation. This metabolic pathway mitigates extracellular matrix degradation and cellular senescence in NPCs. However, another study demonstrated that high-fat diet-induced obesity is associated with LBP but not with structural degeneration of the intervertebral disk (106). Therefore, the relationship between obesity and IDD appears to be complex and multifaceted, warranting further investigation into their potential connections.

Interestingly, Recent studies have intriguingly highlighted the potential involvement of the gut microbiome and its metabolic byproducts in the pathogenesis of IDD. Wang et al. (107) identified that Allobaculum, belonging to the Firmicutes phylum and Clostridia genus, was significantly associated with lipid metabolic products and played a crucial role in IDD development.

## 5 The molecular mechanisms underlying lipid metabolic disorders promoting IDD development

**Figure 3:**
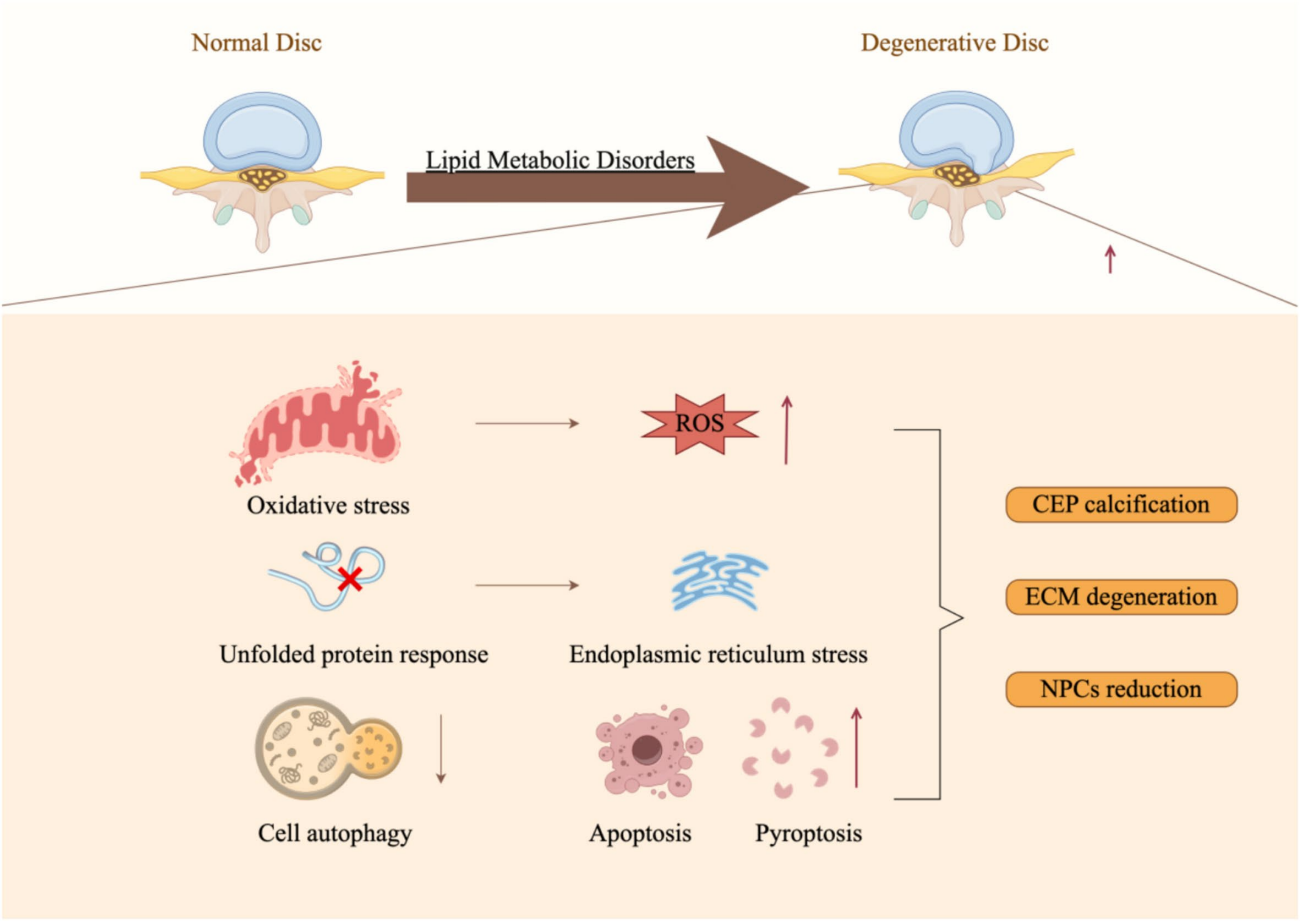
Molecular mechanisms underlying lipid metabolic disorders that facilitate the progression of intervertebral disk degeneration (By Figdraw).

Figure 3 demonstrates that lipid metabolism disorders can lead to a range of detrimental effects, including heightened oxidative stress, induction of endoplasmic reticulum stress, activation of immune cells, pyroptosis, apoptosis, suppression of autophagy, and the promotion of intervertebral disk cartilage calcification through various mechanisms (108), collectively influencing the onset and progression of IDD (Figure 3). These mechanisms are interconnected, forming a complex network that drives the pathological advancement of IDD. Therefore, it is crucial to investigate the underlying mechanisms by which lipid metabolism disorders contribute to IDD to develop novel therapeutic strategies.

### 5.1 Lipid metabolic disorders promote IDD development by activating oxidative stress response

Oxidative stress is the dysregulation between the production of reactive oxygen species (ROS) and the functionality of the antioxidant defense system within biological systems. ROS encompasses a group of highly reactive and unstable chemical entities, which may or may not include free radicals, such as superoxide anion, hydroxyl radical, hydrogen peroxide, and hypochlorite ion (109, 110).

In the pathological progression of IDD, the substantially generated reactive oxygen species (ROS) suggest a correlation between the rate of disk degeneration and elevated ROS (111). Huang et al. revealed that the levels of various oxidative stress biomarkers, such as phospholipase A, fructosamine, malondialdehyde, oxidative potential, total peroxide, advanced oxidation protein products, and nitric oxide (NO), were significantly elevated in the plasma of patients with IDD or in rat models. These elevations may damage DNA, disrupt lipid metabolism, and affect protein synthesis (112). Pathological lipid metabolic disorders trigger the beta-oxidation of fatty acids, resulting in the accumulation of toxic metabolic byproducts that subsequently impair cellular function through various mechanisms, including excessive production of ROS, endoplasmic reticulum stress, and mitochondrial dysfunction (109, 110). Consequently, lipid metabolic disorders and oxidative stress exhibit a significant correlation in IDD.

Lipid metabolic disorders may influence the development or progression of IDD through its interactions with oxidative stress. Previous research has demonstrated that oxidized low-density lipoprotein (ox-LDL) can induce excessive expression of dynamin-related protein 1 (Drp1) and promote the production of mitochondrial ROS, which in turn leads to apoptosis of AF cells and accelerates the progression of IDD (113). Ferroptosis is a newly identified form of regulated cell death, distinguished by dysregulation of iron metabolism, lipid peroxidation, and a Fenton reaction facilitated by free iron ions. In their study, Yang et al. (114) investigated the involvement of ferroptosis in the pathogenesis of IDD. Utilizing an *in vitro* experimental model, the research team noted alterations in the expression levels of ferroptosis markers under oxidative stress conditions alongside increased lipid peroxidation levels. These findings underscore the potential contribution of oxidative stress-induced ferroptosis to the development of IDD. In a recent study, Yao et al. (13) developed an IDD model. They demonstrated that the activity of the transcription factor BACH1 was significantly elevated in IDD tissues derived from rats. The findings indicated that BACH1 plays a crucial role in mediating oxidative stress, iron dysregulation, and lipid metabolism within NPCs by modulating the HMOX1/GPX4 signaling pathway, thereby facilitating the progression of IDD.

Additionally, some researchers have identified iron overload as a significant risk factor for IDD. Wang et al. (79) have elucidated the mechanisms through which iron overload induces oxidative stress and ferroptosis, subsequently promoting calcification of the cartilage endplate via comprehensive experimental investigations. Furthermore, Yang et al. (115) investigated the protective effects of astaxanthin (Ast) on intervertebral disk degeneration-related endplates of the vertebral body and elucidated its potential molecular mechanisms. The study revealed that astaxanthin ECM stability mitigates calcium deposition and reduces apoptosis in CEP cells by activating the Nrf-2/HO-1 signaling pathway, promoting mitophagy, alleviating oxidative stress, and inhibiting ferroptosis in CEP cells. The elevation of tissue stiffness is intricately associated with various pathological processes, including fibrosis, inflammation, and aging. During the degenerative progression of intervertebral disks, the matrix stiffness of NP progressively increases. Research conducted by Ke et al. (116) indicates that in NP cells exhibiting heightened stiffness, there is an upregulation in the expression of Acyl-CoA synthetase long-chain family member 4 (ACSL4), which subsequently facilitates lipid peroxidation and ferroptosis within NP cells.

Moreover, oxidative stress is implicated in the aging of intervertebral disk cells (111), while monoacylglycerol lipase (MAGL) serves as the principal enzyme responsible for the hydrolysis of 2-arachidonoylglycerol (2-AG), facilitating the breakdown of monoglycerides into glycerol and fatty acids. MAGL is pivotal in various pathological processes, including pain, inflammation, and oxidative stress. In this study, Fan et al. employed an NPC aging model induced by lipopolysaccharide (LPS) alongside a rat IDD model induced by needle puncture to investigate the role of MAGL in both *ex vivo* and *in vivo* contexts related to IDD. The findings strongly indicate that inhibiting MAGL can significantly attenuate disk aging through its interaction with STING and may delay the progression of IDD (117).

### 5.2 Lipid metabolic disorders promote IDD development by eliciting an endoplasmic reticulum stress response

Numerous studies have demonstrated that endoplasmic reticulum stress (ERS) is a critical factor in the degeneration of IDD (118–120). Considering that the intervertebral disk is a region of high mechanical stress for protein synthesis, the endoplasmic reticulum (ER) is consequently more vulnerable to the pressures associated with protein synthesis and folding (121). The ER plays a crucial role in preserving the intervertebral disk’s normal physiological structure and function. Endoplasmic reticulum stress (ERS) activates cellular apoptosis and is linked to inflammation, oxidative stress levels, and calcium homeostasis (121). ERS can be triggered by the abnormal accumulation of misfolded or improperly folded proteins, and it is implicated in the pathophysiological mechanisms underlying musculoskeletal disorders, including IDD (118, 122, 123). Notably, the unfolded protein response (UPR) enhances the surface area of the endoplasmic reticulum membrane by upregulating genes associated with lipid metabolism, thereby increasing the capacity of the endoplasmic reticulum (124). Likewise, recent investigations provide additional evidence supporting the intricate relationship between lipid metabolism and the endoplasmic reticulum (125, 126). Previous research has unequivocally demonstrated that cholesterol is integral to the pathogenesis of IDD. In this context, Yan et al. (94) experimentally observed that rat NP cells with exogenous cholesterol exhibited enhanced pyroptosis and ECM degradation. These results further substantiate the pivotal role of ER stress in mediating cholesterol-induced pyroptosis and ECM degradation in NP cells. Chen et al. (127) clarify that LPS activates the endoplasmic reticulum stress (ERS)-C/EBP homologous protein (CHOP) signaling pathway and the ERS-mediated autophagy process, inducing pyroptosis in NPCs and thus delaying the progression of IDD. Bone marrow-derived mesenchymal stem cell exosomes (MSC-exosomes) mitigate endoplasmic reticulum stress-induced apoptosis and enhance IDD recovery by activating the AKT and ERK signaling pathways (122). Hydrogen sulfide (H2S) is a gaseous signaling molecule that has garnered significant attention for its anti-apoptotic properties in various degenerative diseases. The study conducted by Xu et al. further elucidates the role of H2S in mitigating endoplasmic reticulum stress and mitochondrial damage within NPCs, thereby conferring protective effects against IDD (128). Teng et al. conducted a comprehensive investigation into the effects of fucoxanthin (FX) on oxidative stress-induced damage in NP cells, elucidating its potential molecular mechanisms through both *in vivo* and *in vitro* experiments. Their findings indicate that oxidative stress triggers endoplasmic reticulum stress (ERS), apoptosis, and ECM degradation within NP cells (129). Eicosapentaenoic acid (EPA) is an endogenous omega-3 fatty acid present in various plant and animal sources, playing a pivotal role in the growth and development of mammals (130). Prior research has demonstrated that EPA can selectively augment the autophagic activity within cells (131). Building upon this discovery, Lin et al. investigated the mechanisms underlying the role of EPA in IDD. Their experimental findings demonstrate that EPA significantly enhances autophagy activity in NPCs, mitigates endoplasmic reticulum stress, reduces cellular apoptosis, and confers a protective effect on ECM synthesis and degradation. Moreover, *in vivo* studies reveal that EPA ameliorates IDD progression induced by needle puncture in rat models (131).

### 5.3 Lipid metabolic disorders promote IDD development by triggering cellular pyroptosis/apoptosis/inhibiting autophagy

Autophagy is crucial in removing dysfunctional and excessive cellular organelles, such as peroxisomes, mitochondria, nuclei, lysosomes, and ribosomes, ensuring cell survival. During the process of organelle degradation, autophagosomes serve to supply essential nutrients to the cell. The lipid catabolism occurring within the autophagosome/lysosome facilitates the release of fatty acids for mitochondrial oxidation, ultimately leading to the generation of acetyl-CoA (132). The activation of autophagy serves a vital protective role in IDD by promoting cell survival and inhibiting apoptosis, thereby underscoring its fundamental function in cellular protection (133). Furthermore, autophagy-mediated cell death, a distinct form of autophagic cell death that operates independently of other cell death modalities, has been unequivocally characterized (134).

Recent reports indicate a significant interplay between lipid metabolism and autophagy. Li et al. (135) demonstrate that starvation-induced autophagy markedly influences lipid metabolism, encompassing free fatty acid, glycerophospholipid, and sphingolipid metabolism. These findings elucidate the pivotal role of autophagy in lipid metabolic processes, particularly its regulatory function in energy production and autophagosome formation, as well as its implications for cellular protection and apoptosis under nutritional stress conditions. Cheng et al. (136) explored how damage caused by partner-mediated autophagy (CMA) in the degradation process of phospholipase Cγ1 (PLCG1) promotes cell aging and IDD through the regulation of CMA on intracellular calcium flux. The study confirms that PLCG1 is a key mediator of CMA in regulating intracellular calcium flux in cells. Due to the blockage of CMA, PLCG1 accumulates abnormally, leading to calcium overload and inducing cellular senescence of NPCs, thus causing IDD. Dan et al. (137) demonstrated that the overexpression of ARRB1 inhibits apoptosis and extracellular matrix degradation in rats, enhances autophagy in NPCs, and attenuates the progression of IDD. Li et al. (138) found that compressive stress can induce autophagy in NPCs through the modulation of the PI3K/AKT/mTOR signaling pathway associated with ROS, subsequently activating the JNK signaling pathway, thereby ameliorating IDD induced by compressive stress. Zhang et al. (139) established a model of chondrocyte endplate stem cell (CESC) degeneration induced by tensile loading in a large rabbit model experiment in 2023. They found that inhibiting the expression of JNK and ERK could suppress the phosphorylation of Raptor and mTOR, thereby improving autophagy levels in the mTOR signaling pathway and alleviating the degradation of CESC.

Generally, autophagy is intricately linked to the mechanisms of cell apoptosis and cell death (140, 141). A complex interplay exists between autophagy and apoptosis, characterized by the involvement of various shared regulatory factors and signaling pathways (142). Irisin is a myokine released from the membrane-bound precursor protein fibronectin type III domain-containing protein 5 (FNDC5) following proteolytic cleavage, and its levels are elevated in response to physical exercise (143); as an exercise-induced myokine, it facilitates lipid metabolism (144, 145). Based on these results, Zhou et al. (146) investigated the role of FNDC5/irisin in mediating the effects of physical activity on IDD. For the first time, they confirm from a muscle-derived factor perspective that physical activity modulates autophagy levels within the NP of intervertebral disks via FNDC5/irisin, thereby mitigating the progression of IDD. Wang et al. (147) demonstrated that KuA (salidroside) mitigates neuronal cell death, ECM deposition, and inflammatory responses induced by LPS in NPCs through the activation of the phosphatidylinositol 3-kinase/protein kinase B (PI3K/Akt) signaling pathway, thereby contributing to the alleviation of IDD.

Pyroptosis, a recently identified form of inflammatory programmed cell death occurring in macrophages, is contingent upon the activation of caspase-1. Beyond its role in significantly diminishing immune cell populations, pyroptosis can also incite excessive inflammatory responses within the organism, resulting in tissue and organ damage and potentially culminating in mortality (148, 149). The study conducted by Wu et al. unequivocally established a direct correlation between pyroptosis and ferroptosis in the progression of IDD. Their findings reveal that glutamine facilitates the deubiquitination of Nrf2 while concurrently inhibiting lipid oxidation in NPCs, thereby effectively mitigating cellular processes induced by oxidative stress, including pyroptosis, ferroptosis, and ECM degradation (150). Recent investigations by scholars have elucidated the role of EZH2-H3K27me3-mediated epigenetic silencing in the activation of NLRP3 and NAIP/NLRC4 inflammasome pathways, thereby inducing pyroptosis in NPCs (151). Similarly, Yu et al. demonstrated that PR/SET domain 1 (PRDM1) expression was markedly elevated in degenerated NP and NPCs. Furthermore, they found that the overexpression of PRDM1 intensifies pyroptosis in NPCs, thereby facilitating the progression of IDD (152).

## 6 Conclusion

Lipid metabolic disorders are widely recognized as a contributing factor to the onset and progression of various diseases, including cancer, type 2 diabetes mellitus (T2DM), and osteoarthritis (69). Jin et al. (153) employed Mendelian randomization analysis to evaluate the relationship between T2DM and IDD. Their study found that, even after adjusting for body mass index (BMI), T2DM remains a causally related risk factor for IDD. According to previous studies, T2DM induced by leptin receptor knockout can lead to IDD through increased MMP3 levels and exacerbated cellular apoptosis (154). Ding-Qiang et al. (155) utilized Mendelian randomization (MR) analysis in conjunction with genome-wide association study (GWAS) data to investigate the causal relationship between T2DM and IDD, as well as to quantify the mediating effect of triglycerides in this association. The results demonstrated that T2DM increases the risk of IDD, with a portion of this increased risk mediated by TG. Specifically, TG accounts for 11.4% of the effect of T2DM on IDD.

In addition, lipid metabolic disorders contribute to the progression of atherosclerosis, resulting in diminished blood flow to the lumbar region and consequently heightening the risk of IDD, sciatica, and lower back pain (70, 71). A recent study examined intervertebral disk cells obtained from patients with IDD and healthy subjects, revealing that the levels of various lipid metabolites—including triacylglycerol, diacylglycerol, fatty acids, phosphatidylcholine, lysophosphatidylinositol, and sphingomyelin—were significantly reduced in degenerated intervertebral disk cells. Conversely, the levels of bile acids and ceramides were found to be elevated. These findings suggest a metabolic shift in intervertebral disk cells from glycolysis toward fatty acid oxidation, which may ultimately contribute to cell death and facilitate the progression of IDD (156). As mentioned above, Kaye et al. (112) observed that the levels of various oxidative stress biomarkers—such as phospholipase A, fructosamine, malondialdehyde, peroxidation potential, total hydrogen peroxide, advanced oxidation protein products, and NO—were significantly elevated in the plasma of patients with IDD or in rat models. These alterations may contribute to DNA damage and disorders in lipid metabolism and protein synthesis. Yan et al. (94) conducted experiments that demonstrated rat NP cells treated with exogenous cholesterol exhibited accelerated pyroptosis and degradation of ECM. These findings underscore the critical role of endoplasmic reticulum stress in mediating cholesterol-induced pyroptosis in NP cells, as well as the subsequent degradation of ECM. The findings of Dan et al. demonstrated that the overexpression of ARRB1 can inhibit apoptosis and extracellular matrix degradation in rat cells, promote autophagy in NPCs, and delay the progression of IDD (137). Zhang et al. (139) established a model of chondral endplate stem cell (CESC) degeneration induced by tensile load in a large white rabbit model. Their findings indicate that the inhibition of JNK and ERK expression can suppress the phosphorylation of Raptor and mTOR, thereby enhancing the autophagy levels of CESC within the mTOR signaling pathway and mitigating its degradation. In summary, Lipid metabolic disorders primarily contribute to the degeneration of CEP, NP and other structures through the activation of inflammatory responses, which in turn trigger endoplasmic reticulum stress, elevate oxidative stress levels, promote cell death and pyroptosis, and inhibit autophagy. These interconnected mechanisms form a complex network that collectively drives the pathological progression of IDD. The significance of lipid metabolism in IDD has garnered increasing attention within the academic community. A deeper understanding of lipid metabolism’s role in IDD elucidates the underlying mechanisms of lumbar intervertebral disk degenerative diseases. It offers novel strategies and targets for their prevention and treatment. For instance, Wang et al. (97) developed a natural hydrogel composite that ameliorates IDD by modulating fatty acid metabolism and inhibiting pyroptosis in NPCs. A cross-sectional observational study investigated the relationship between lipid-lowering medications and degenerative disk disease, sciatica, and LBP, revealing a significant positive correlation between the non-use of statins and the incidence of LBP (157). Zhang et al. (158) substantiated this perspective, demonstrating that rosuvastatin is a promising therapeutic agent for ameliorating IDD.

Lipid metabolites have the potential to serve as biomarkers for intervertebral disk injury, offering novel insights for early diagnosis and treatment. Nevertheless, current research faces several limitations: the pathological mechanisms underlying IDD are exceedingly complex, encompassing genetic, biomechanical, and cellular biological factors, among others. Lipid metabolism represents merely one facet of this intricate process. Our understanding of how lipid metabolism interacts with these various factors remains limited. Many studies predominantly utilize animal models or *in vitro* cell experiments that may not adequately replicate the complexities inherent to human IDD. Furthermore, given that IDD is a protracted developmental process, numerous investigations lack long-term follow-up data; thus, our comprehension of the enduring effects of lipid metabolic disorders on IDD is constrained. Future research should prioritize interdisciplinary collaboration and comprehensive approaches to elucidate fully the pathological mechanisms associated with IDD and devise holistic prevention strategies. Through such collaborative efforts across disciplines, significant advancements in preventing and treating IDD are anticipated.
